# Does Blindness Boost Working Memory? A Natural Experiment and Cross-Cultural Study

**DOI:** 10.3389/fpsyg.2020.01571

**Published:** 2020-07-03

**Authors:** Heiner Rindermann, A. Laura Ackermann, Jan te Nijenhuis

**Affiliations:** ^1^Department of Psychology, Chemnitz University of Technology, Chemnitz, Germany; ^2^Experimental and Applied Psychology, Vrije Universiteit Amsterdam, Amsterdam, Netherlands

**Keywords:** working-memory capacity, intelligence, cognitive ability, modifiability, blindness, cross-cultural comparison, natural experiment

## Abstract

Intelligence requires sufficient working-memory capacity. Traditionally, working memory was seen as a process and as a prerequisite for fluid intelligence. Working memory was assumed to be determined by maturation and health. There is a gap in the literature: It is still not fully understood to which extent and how working memory can be influenced. So this study tested how visual impairment and the extent of visual impairment are related to working memory capacity. In our study we compared *N* = 249 children (6–16 years) with and without visual impairment (blind, visually impaired, and sighted) in two countries (South Africa and Austria) at different development levels on their working-memory capacity and verbal comprehension. Using the WISC-IV, blind and visually impaired children showed higher working-memory capacity than sighted children (*r* = + 0.35, 14, and 3 IQ points, respectively). On the other hand, visually impaired children showed a weakness in verbal comprehension (*r* = −0.39, on average 13 IQ points lower). The pattern remained robust when SES and race-ethnicity were controlled. Our natural (quasi-)experiment shows a pattern, which is unlikely to be genetic, and so supports the view that working memory and intelligence scores can be modified.

## Introduction

### Changeability of Cognitive Ability

A question that has been hotly debated for a long time in intelligence research is the changeability of cognitive ability. On the one hand, studies showed a long-term stability of individual differences in IQ across the lifetime, e.g., from age 11 to age 77 years *r* = 0.63 (Deary et al., 2000), and training and intervention studies frequently showed small effects or effects that faded out (Protzko, 2015). On the other hand, there is a huge increase in intelligence in youth due to learning in school and there is a decrease in old age (e.g., Cattell, 1987/1971; Rindermann, 2011; Ritchie and Tucker-Drob, 2018). While knowledge and specifically crystallized intelligence are generally seen as modifiable by learning and environment, fluid intelligence (culture-reduced reasoning) and especially basic cognitive processes and competences, such as mental speed and working memory, are generally considered as hardly changeable.

We define intelligence as the ability to think: (1) to solve new problems by thinking, (2) to inductively and deductively infer, (3) to think abstractly, and (4) to categorize and to understand. Intelligence is an essential precondition for academic performance and professional success (e.g., Gottfredson, 2003; Hunt, 2011). Together with mental speed, working memory is a basic cognitive process in thinking (Jensen, 2006; Rindermann et al., 2011). Mental speed is needed to quickly process information. Working memory is the ability to simultaneously compare and store different information in short-term memory (Baddeley, 2007). Working-memory capacity – a stable individual differences variable – is highly correlated with intelligence: Various researchers found correlations at around *r* = 0.50 (Ackerman et al., 2005); in a study by Kyllonen and Christal (1990), the correlations even reach a value of *r* = 0.80–0.90. These values are much higher than the usual values of the correlations between mental speed and IQ (around *r* = 0.20–0.30; Jensen, 2006).

As intelligence scores are excellent predictors of school achievement and job performance, the question whether working memory can be effectively trained is of great scientific and practical importance. Jaeggi et al. (2008) developed a working-memory training program and the authors concluded that the outcomes showed positive effects on working-memory capacity and intelligence, but these conclusions were not generally accepted (e.g., Moody, 2009; Redick et al., 2013; Melby-Lervåg et al., 2016). While some meta-analytic evidence strengthened Jaeggi’s position (e.g., the average training effect across 24 measures on fluid intelligence was *d* = 0.24; Au et al., 2015), other meta-analyses reported less supportive outcomes (e.g., *d* = 0.08–0.15 for IQ; Melby-Lervåg et al., 2016). Some authors of meta-analyses argued in particular, that cognitive training programs such as on working memory do not sustainably improve overall cognitive performance (i.e., fluid intelligence), but only the performance on certain working-memory tasks (e.g., Protzko, 2017; especially for children see Simons et al., 2016; or Takacs and Kassai, 2019).

However, looking at direct effects on working memory itself, the training effects are more favorable, e.g., on verbal working memory *d* = 0.31–0.42, on visuospatial working memory *d* = 0.28–0.51, and on specific study criterion measures *d* = 0.80–1.88 (Melby-Lervåg et al., 2016, their Table 1). These findings are corroborated by a second meta-analysis by Soveri et al. (2017): the effect on working memory measured using n-back tasks was *d* = 0.62, on other working-memory tasks *d* = 0.24, but on fluid intelligence *d* = 0.16. So, it is clear that working memory itself benefits from working-memory training.

**TABLE 1:** Sample characteristics for the South African and the Austrian sample.

	Gender	Age	Social status	
Sample	(*N* = 249)	(*N* = 249)	(*N* = 212)	
South Africa	Male: *N* = 77 (49.7%)	*M* = 13.38	SES1 (township)	*N* = 35 (22.6%)
(*N* = 155)	Female: *N* = 78 (50.3%)	*SD* = 2.18	SES2 (lower class)	*N* = 62 (40.0%)
			SES3 (middle class)	*N* = 58 (37.4%)
			SES4 (upper class)	*N* = 0 (0.0%)
Austria	Male: *N* = 45 (47.9%)	*M* = 12.34	SES1 (township)	*N* = 1 (1.1%)
(*N* = 94)	Female: *N* = 49 (52.1%)	*SD* = 3.58	SES2 (lower class)	*N* = 7 (7.4%)
			SES3 (middle class)	*N* = 44 (46.8%)
			SES4 (upper class)	*N* = 5 (5.3%)

*N_(social status)_ ≠ [N_(South Africa)_ + N_(Austria)_] because not all participants provided information on their social status.*

### Blindness as a Natural Experiment to Test Changeability

Nevertheless, there still remains an important theoretical objection as working memory is seen as being determined by maturation and health. Working-memory capacity increases in childhood and declines in old age, suggesting a strong biological cause (e.g., Salthouse, 1990). A different approach can help determine to what degree working memory is alterable: We used data from a natural experiment (Dunning, 2012), a quasi-experiment “in the field,” looking at children with a visual disability – the visually impaired and even completely blind persons – as they depend much more than the sighted on their working memory to process information. While solving cognitive tasks these persons cannot draw on external visual representations of information, such as texts or figures. Hence, persons with a visual disability have to rely particularly on *internal* representation, storage, and processing of information and this is more demanding of working memory. So, they need to compensate for not having the possibility of external storage for information (e.g., written texts, figures, tables, or graphs). If working-memory capacity is changeable through experience, it seems logical to assume that the more intensive use of working memory due to a visual impairment should boost working-memory capacity (e.g., Hupp, 2003). Furthermore, research suggests that a form of brain plasticity can also lead to superior working memory. Various studies observed that blind persons can use additional brain resources which are otherwise invested in processing visual input to enhance cognitive abilities in other domains (Röder et al., 2002; Bedny et al., 2015; Abboud and Cohen, 2019). Amedi et al. (2003), for instance, indicated that superior verbal memory performance in blind subjects could be due to activation of the cortical visual system. Other authors such as Kattner and Ellermeier (2014) showed that blind people are more resistant to irrelevant auditory speech-like stimuli in regard to their working-memory performance (Kattner and Ellermeier, 2014).

Additionally, certain educational aids such as braille, auditory explanation of pictures and graphs, and auditory books may further enhance working-memory capacity. Following the same reasoning, because all access to printed information and to visual information is limited for persons with visual impairment, they are expected to have less extensive knowledge. Some studies already hinted that compared to non-blind persons blind persons have some advantage on working-memory tasks (e.g., Tillman and Osborne, 1969; Smits and Mommers, 1976; Hull and Mason, 1995; Hupp, 2003; Withagen et al., 2013; Pigeon and Marin-Lamellet, 2015), and that participants with a visual disability may perform worse on knowledge-based scales (e.g., Wyver and Markham, 1999; Hupp, 2003). The results of the studies mentioned are in line with the assumption that visual disability acts like a training for working memory but at the same time impedes the acquisition of crystallized intelligence. In this context, several neuropsychological studies have addressed the hypothesized association between working memory and sentence comprehension (e.g., Varkanitsa and Caplan, 2018). There is an ongoing debate whether working memory and sentence comprehension are connected to the same or different neuronal systems (e.g., Pisoni et al., 2019).

### Hypotheses

Four hypotheses were tested using two samples from highly diverging environments, namely Africa and Europe:

(1) Children with a visual disability have better working-memory capacity (WMC) than sighted children.
(2) Children with a visual disability have lower verbal comprehension than sighted children.
(3) The more serious the visual disability, the better the working-memory capacity and the lower the verbal comprehension (dose-response relationship).
(4) Blind-sighted differences in working-memory capacity and verbal comprehension (VC) should be environmentally caused (based on experience as a person with a visual disability).

## Materials and Methods

### Design and Eyesight

We used the natural experiment of blindness to test these hypotheses and compared sighted, visually impaired, and blind children in two countries at different developmental levels. Eyesight was determined by visual acuity (VA). A person’s visual acuity is reported as a quotient: It is the distance where that person can visually recognize a certain stimulus, divided by the average distance where this stimulus normally can be visually recognized. In practice, stimuli of different sizes (mostly letters on a board) are presented to the participant while he or she sits at a certain fixed distance from the stimuli (for a more detailed description see Kniestedt and Stamper, 2003). Visual acuity was always tested under optimal conditions (best eye, with vision aid).

Visual ability (eyesight, degree of visual impairment) was the independent variable, and it consisted of three categories: blind (VA < 1/20), visually impaired (VA of 3/10–1/20), and sighted (VA of >3/10 or visual field in case of central fixation is less than 5°). This classification was taken from the 10th revision of the International Statistical Classification of Diseases and Related Health Problems (ICD-10). As dependent criteria, working-memory capacity and verbal comprehension were measured.

Further possible determinants and biasing factors, such as SES, race-ethnicity, gender, age, and age of onset of visual impairment were measured.

### Sample

As mentioned before, the meta-analyses of Melby-Lervåg et al. (2016) and Soveri et al. (2017) have shown that working-memory capacity is changeable by training (depending on criteria and control groups with effects between *d* = 0.24 and 1.88). Because a visual impairment can influence fluid intelligence and its components much longer and stronger than a temporary exceptional training, we expected a medium effect size of *d* = 0.50 between children with and without visual disability regarding their working-memory capacity. To ascertain adequate power of 95% to find this effect (assuming a conventional error probability of α = 0.05), a sample size of 132 children with and 66 children without visual disabilities was recommended. *De facto* we were able to recruit a total of 153 children with visual disabilities and 96 children without visual limitations.

The South African sample was from Cape Town and Worcester, situated 120 km from Cape Town, and consisted of 155 children (mean age 13.38 years, range 6–16 years). 45 were blind, 58 were visually impaired, and 52 had no visual handicaps (sighted). 77 were boys, 78 girls. 35 children had the lowest social-economic status (SES), 62 had low SES, 58 had medium SES, and none had high social status. 64 were Black, 52 Colored (the conventional term in South Africa), and 39 White. 26 spoke English, 66 Afrikaans, and 63 Xhosa as their first language. SES was measured according to criteria developed by Statistics South Africa (2000): accommodation (informal vs. formal settlement), home equipment (running water, electricity, bathroom, and telephone), highest educational degree of parents and of further adults living in the home, number of persons per room, and family income. The same measure was applied to the Austrian sample.

The Austrian sample was from, respectively, the province of Styria and the Austrian capital Vienna, and consisted of 94 children (mean age 12.35 years, range 6–16 years). 19 were blind, 31 were visually impaired, 44 had no visual handicaps (sighted). 45 were boys, 49 girls. One child had the lowest social status, seven low SES, 44 medium SES, and five high social status (rest missing data). All were White. 85 spoke German, five Serbo-Croatian, and four Turkish as their first language. Table 1 gives information on gender, age, and social status.

Table 2 gives an overview of the groups of blind, visually disabled and sighted participants of both (South African and Austrian) samples. In both samples, the blind as well as the children with a visual disability went to special schools. Those schools use didactical techniques adjusted to students with severely limited or no visual ability and were specialized in teaching braille. But the curricula of the blind and the students with visual disability in our sample was still broadly similar to those of general public schools, e.g., both teaching mathematics, language, and history. In our sample of participants with visual disability, no students with multiple disabilities were included. The blindness or the visual disability in our samples had various causes and was either congenital (*N* = 114) or acquired (*N* = 39).

**TABLE 2:** Sample characteristics for the participants with and without visual disability.

	Gender	Age	Social status	
Sample	(*N* = 249)	(*N* = 249)	(*N* = 212)	
Blind	Male: *N* = 25 (39.1%)	*M* = 13.21	SES1 (township)	*N* = 11 (20.4%)
(*N* = 64)	Female: *N* = 39 (60.9%)	(*SD* = 2.67)	SES2 (lower class)	*N* = 22 (40.7%)
			SES3 (middle class)	*N* = 20 (37.0%)
			SES4 (upper class)	*N* = 10 (1.9%)
Visually impaired	Male: *N* = 50 (56.2%)	*M* = 13.33	SES1 (township)	*N* = 15 (20.8%)
(*N* = 89)	Female: *N* = 39 (43.8%)	(*SD* = 2.68)	SES2 (lower class)	*N* = 27 (37.5%)
			SES3 (middle class)	*N* = 30 (41.7%)
			SES4 (upper class)	*N* = 0 (0.0%)
Sighted	Male: *N* = 47 (49.0%)	*M* = 12.52	SES1 (township)	*N* = 10 (11.6%)
(*N* = 96)	Female: *N* = 49 (51.0%)	(*SD* = 3.03)	SES2 (lower class)	*N* = 20 (23.3%)
			SES3 (middle class)	*N* = 52 (60.4%)
			SES4 (upper class)	*N* = 4 (4.7%)

*N_(social status)_ ≠ [N_(blind)_ + N_(visual. handic.)_ + N_(sighted)_] because not all participants provided information on their social status.*

### Procedure

The selection of the participants was as follows: in South Africa, two institutions especially for the blind (“Athlone School for the Blind” in Cape Town and “Pioneer School” in Worcester) were contacted to get access to a larger group of children with visual disabilities. Additionally, public schools in Cape Town and Worcester with special integration classes for children with visual disabilities were contacted to acquire children with visual disabilities. To generate a corresponding, representative sample of children with no visual limitations, regular public schools and community centers in Cape Town and Worcester were randomly selected and asked to participate. After the various institutions confirmed their willingness to support the study, the students (and their parents) decided voluntarily whether they wanted to participate in the study.

In Austria, the procedure was quite similar: the Odilien-Institut in Graz (province of Styria) and the Federal Institute for the Blind in Vienna had been contacted to acquire an adequate sample of children with visual disabilities. In addition, general public schools in Graz and Vienna were randomly contacted to generate an adequate sample of sighted children. After the institutions confirmed their willingness to participate, the students and their parents voluntarily assented to the study. The working-memory tests and the verbal ability tests were administered in the respective schools as group tests. Only some of the South African participants (single blind students at the Pioneer school and the public schools with integration classes) had to be tested at home due to time constraint and organizational limitations.

### Tests

Working-memory capacity (WMC) and verbal comprehension were measured with subtests of the Wechsler Intelligence Scale for Children IV (WISC-IV; Wechsler, 2003). Both scales can be administered without using paper and pencil. The South African sample was directly tested using the original English version of the test, whereas the German translation of the WISC-IV was used for the Austrian sample (Hamburg-Wechsler-Intelligenztest für Kinder, HAWIK-IV; Petermann and Petermann, 2007, 2011).

The compound score of *working memory* combines the scores on two subtests (1) digit span (repeating numerical series in proper or reverse order) and (2) letter-number sequencing (repeating a given set of numbers and letters in numerical or alphabetical order). A third subtest, measuring arithmetic (solving mental-calculation tasks that are orally presented), is less appropriate and is only supplemental (e.g., it can be used if one of the main subtests cannot be applied; Petermann and Petermann, 2011, p. 16). In the current study, the third subtest was taken but only used for the *g*-factor analysis.

The compound score of *verbal comprehension* combines the scores on three subtests: (1) similarities (finding a hypernym for a given set of words), (2) vocabulary (defining a given word), and (3) comprehension (knowledge about social policies and everyday problems). The supplemental subtest information (knowledge about public events, issues, and persons) was also taken, but because none of the regular subtests had to be replaced, it was used only for the *g*-factor analysis.

To combine the results of the South African and Austrian samples, the results on both subtests were standardized using the norm values of the German HAWIK-IV. As usual, subtests were age-normed, so the mean age difference of 1 year between the South African and Austrian students had no influence.

### Analyses

We compared for each of the two samples (South Africa and Austria) and within each of the three groups (the blind, visually impaired, and sighted) averages in working-memory capacity and verbal comprehension. We computed means and standard deviations, correlations (Pearson and Spearman for robustness checks) with eyesight, differences in IQ points (using the standard deviations of the nationally representative samples), and differences in standard deviations using sample *SDs*. Significance tests were not used for interpretation (for an in-depth justification, see, e.g., Cohen, 1994; Gigerenzer, 2004; Wasserstein et al., 2019).

In regression analyses we checked the robustness of the visual impairment effect for country (South Africa “0” vs. Austria “1”), SES (township “1,” lower class “2,” middle class “3,” and upper class “4”) and ethnicity (race; black “0,” colored “1”, and white “2”).

Finally, we performed two *g*-factor analyses in an attempt to answer the following questions: are eyesight differences larger on the two working-memory capacity and verbal comprehension *g* factors than on the two mean scales? Are *g*-factor loadings correlated with the subtest-eyesight correlations? If blind-sighted differences are larger on *g* factors and if these differences are correlated with *g*-factor loadings this would hint to a genetic causation; if they are not larger on *g* factors and not correlated with *g*-factor loadings this suggests an environmental causation (te Nijenhuis and van der Flier, 2013). Due to the small number of subtests (*k* = 3 WM subtests and *k* = 4 VC subtests) these analyses are exploratory.

## Results

**FIGURE 1:**
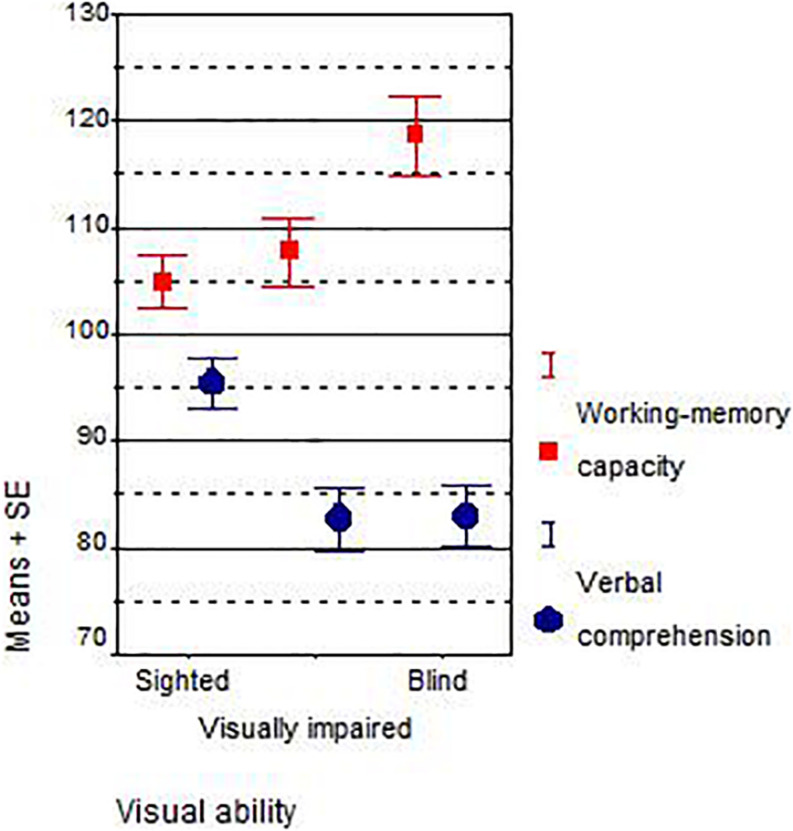
Main effects dimension × visual ability.

The average results in working-memory capacity and verbal comprehension of the blind, visually impaired, and sighted South African and Austrian children are presented in Table 3 and Figure 1.

**TABLE 3:** Average WMC and verbal comprehension scores depending on visual ability.

Sample	Blind	Visually impaired	Sighted	*r* [95% CI]	Cohen’s *d* [95% CI]
	*M* (*SD*)	*M* (*SD*)	*M* (*SD*)		
**South Africa (*N* = 155)**					
WMC	120.47 (10.47)	110.43 (12.94)	103.21 (10.14)	+0.52 [+0.39, +0.63]	+1.67 [+1.42, +1.93]
VC	80.16 (10.82)	78.53 (11.71)	92.06 (8.68)	−0.40 [−0.52, −0.26]	−1.22 [−1.45, −0.97]
**Austria (*N* = 94)**					
WMC	114.00 (21.35)	102.58 (17.29)	106.77 (14.10)	+0.11 [−0.09, +0.31]	+0.40 [+0.17, +0.62]
VC	89.63 (9.44)	90.29 (13.55)	99.50 (12.69)	−0.33 [−0.49, −0.13]	−0.89 [−1.12, −0.65]
**All together (*N* = 249)**					
WMC	118.55 (14.69)	107.70 (14.98)	104.84 (12.18)	+0.35 [+0.24, +0.45]	+1.02 [+0.78, +1.25]
VC	82.97 (11.24)	82.63 (13.53)	95.47 (11.28)	−0.39 [−0.49, −0.28]	−1.11 [−1.35, −0.87]

*WMC, working-memory capacity; VC, verbal comprehension; r, correlation between visual ability (sighted: “0,” visually impaired: “1,” blind: “2”) and ability results in the respective IQ dimension; d = intelligence difference between the blind and sighted expressed in standard deviation units (M = 0, SD = 1) using the averaged standard deviations of the blind and sighted of this sample (not SD = 15), positive values mean higher results for the blind.*

Blind students outperformed sighted students on working memory, both in South Africa (*r* = +0.52, Spearman *r*_S_ = +0.54, IQ +17, *d* = +1.67) and Austria (*r* = +0.11, *r*_S_ = +0.04, IQ +7, *d* = +0.40). As predicted, blind students scored worse on verbal comprehension than sighted students and this is true for both the South African (*r* = −0.40, *r*_S_ = −0.43, IQ −12, *d* = −1.22) and Austrian sample (*r* = −0.32, *r*_S_ = −0.36, IQ −10, *d* = −0.89). Within the subsamples of visual disabilities, the trend is monotonous only for the working-memory capacity of South African students: the more strongly the visual disability was, the better were the working-memory test scores (from IQ 103.21–110.43 and 120.47). A weak trend could be found for the scores on verbal comprehension of Austrian students: the better the eyesight, the higher their verbal comprehension (from IQ 89.63–90.29 and 99.50). Across all groups and all samples, the results on the verbal comprehension subtest were lower than the results on working memory. The overall correlation between both scales, working memory and verbal comprehension, was *r* = 0.22, which is not high.

Checking the effects at the level of subtests of working memory, visual impairment was positively correlated with digit span (for all three groups of eyesight: *r* = +0.40, South Africa: *r* = +0.60, Austria: *r* = +0.11) and letter-number sequencing span (*r* = +0.20, South Africa: *r* = +0.24, Austria: *r* = +0.15) separately, for working memory (mean score) the correlations were *r* = +0.35 (*r*_S_ = +0.37; South Africa: *r* = +0.52, *r*_S_ = +0.54, Austria: *r* = +0.11, *r*_S_ = +0.04).

There were only minor or unsystematic differences between the general cognitive ability levels (WM and verbal comprehension averaged) of the blind (*M* = 100.76, *SD* = 10.79), impaired (*M* = 95.16, *SD* = 11.91) and sighted (*M* = 100.16, *SD* = 9.83). Blindness does not necessarily lead to reduced intelligence, particularly not for children attending special schools for students with visual disability, as is the case for both our samples. There are also only small differences between South African (*M* = 97.23, *SD* = 9.63) and Austrian (*M* = 100.66, *SD* = 12.95) students. This is at odds with the generally found large gap between Western and sub-Saharan African samples (Rindermann, 2018). However, on closer inspection the South African data reveal a racial-ethnic gap of around 14 IQ points that is stable across all three groups of eyesight. The South African Whites’ average IQ of 105.78 (*SD* = 5.02) suggests that the South African sample was positively selected. The Austrian sample seems not to be selected (grand mean IQ 100), but the same ability pattern for eyesight was found (i.e., blind vs. sighted and working memory vs. verbal).

Visual impairment (sighted “0,” visually disabled “1,” blind “2”) was not correlated with country, gender, social status, ethnic background, and first language (all correlations below *r* = 0.15). At best, there was a small negative correlation between visual impairment and SES (*r* = −0.13 and −0.12 in South Africa and Austria, using Spearman’s rank correlation *r*_S_ = −0.13 and −0.18). However, country and SES (*r* = 0.44, *r*_S_ = 0.45) were substantially correlated. Within South Africa, social status and racial-ethnic background were highly correlated with verbal comprehension (*r*_SES_ = 0.61, *r*_Ra_ = 0.62, *r*_S_ = 0.64 and 0.62, respectively), and also SES with race-ethnicity (*r* = 0.71, *r*_S_ = 0.71).

Gender was not correlated with eyesight (*r* = 0.06), WMC (*r* = 0.09), and VC (*r* = −0.01). Age was weakly correlated with eyesight (*r* = 0.11), WMC (*r* = 0.05), and VC (*r* = −0.23). The negative correlation between age and verbal comprehension (*r* = −0.23, South Africa *r* = −0.04, Austria *r* = −0.31) showed no systematic covariation with eyesight (e.g., in Austria for the sighted higher, in South Africa for the blind even weakly positive).

Age of onset of visual impairment (with birth/congenital 0, later 1) was not correlated with eyesight (impaired or blind, *r* = 0.02) and only weakly with WMC (*r* = 0.05) and VC (*r* = −0.14). That means persons with later-developed visual impairment had a slightly lower verbal comprehension compared to those with congenital visual impairment. Blind persons suffer more from later visual impairment (for the visually disabled onset and WM *r* = 0.15, onset and VC *r* = −0.06; for the blind onset and WM *r* = −0.12, onset and VC *r* = −0.28).

We checked whether the visual impairment (VI) effect on WM and VC depends on other factors (country/“Co”, SES, race/“Ra”). In common analyses (both countries together) on WMC, visual impairment showed the strongest effect (a positive one): β_Co__→__WMC_ = −0.14, β_SES__→__WMC_ = 0.16, β_Ra__→__WMC_ = 0.23, and β_VI__→__WMC_ = 0.49. Within South Africa the results are similar (β_SES__→__WMC_ = 0.18, β_Ra__→__WMC_ = 0.21, and β_VI__→__WMC_ = 0.55), also within Austria (β_SES__→__WMC_ = 0.06 and β_VI__→__WMC_ = 0.31). For verbal comprehension (VC) the effect of visual impairment is also robust (a negative one): β_Co__→__VC_ = 0.05, β_SES__→__VC_ = 0.24, β_Ra__→__VC_ = 0.40, and β_VI__→__VC_ = −0.30. Within South Africa the results are similar (β_SES__→__VC_ = 0.29, β_Ra__→__VC_ = 0.40, and β_VI__→__VC_ = −0.34), also within Austria (β_SES__→__VC_ = 0.15 and β_VI__→__VC_ = −0.29). For working memory, eyesight is always the most important variable. However, for verbal comprehension race-ethnicity reveals to be even more important than country, SES or eyesight, but there is still a stable negative impact of visual impairment (β_VI__→__VC_ = −0.30, −0.34, and −0.29). Remarkable is also the small country effect when controlled for SES and race (and eyesight). In the Supplementary Material, the analyses are also repeated now using a *g* factor score instead of scale means.

## Discussion

Previous meta-analyses have shown that working-memory capacity is changeable by training (depending on criteria and control groups between *d* = 0.24 and 1.88; Melby-Lervåg et al., 2016; Soveri et al., 2017). Our results using a natural experiment comparing visually impaired with sighted persons, shows even larger effects. Blind and sighted and visually impaired and sighted persons in two countries differ in working memory: the blind score 14 IQ points higher and the visually impaired score three IQ points higher, whereas the opposite pattern was found for verbal comprehension.

Our conclusion is backed by several studies that came to similar results (Tillman and Osborne, 1969; Smits and Mommers, 1976; Hull and Mason, 1995; Hupp, 2003; Withagen et al., 2013; Pigeon and Marin-Lamellet, 2015): blindness seems to raise working memory. Of course, it is *not blindness itself*, but the individual and institutional reaction to blindness compensating for a shortage in visual information and in external visual storage of information.

The blind and sighted are quite similar in total IQ (average of working memory and verbal comprehension combined). This might be caused by the special schools for the blind attempting to compensate for the negative effects of serious deprivation by supplying specially tailored cognitive stimulation (e.g., the usage of special learning materials or technical devices, additional learning opportunities within working group).

Regressions with country, with SES, and with race-ethnicity controlled for, show a robust effect of visual impairment. Nevertheless, eyesight is not the only relevant factor explaining individual differences in working memory and verbal comprehension. While gender was not correlated with the two cognitive measures, social status and race-ethnicity showed a correlation, especially with verbal comprehension (for the total sample *r* = 0.62, *r* = 0.58), the correlation with country was smaller (*r* = 0.39). The correlations were higher in South Africa than in Austria (social status and VC: *r*_ZAF_ = 0.60 vs. *r*_AUT_ = 0.19).

## Limitations

In our study, WMC and verbal comprehension were both measured using the WISC-IV. This test was often used in studies on the changeability of WMC (e.g., Au et al., 2015) and enabled us to measure both abilities in a valid and economic way (Kaufman et al., 2006). Additionally, the WISC-IV is available in comparable versions in English and German and therefore can be directly administered in both of our samples. However, the WISC-IV is measuring only three facets of WMC (digit span, letter-number sequencing and the supplemental facet arithmetic). So further research should check the results of this study by measuring WMC in a more non-verbal way (e.g., analogous block span tests or the self-ordered pointing test; see Baron, 2005).

The trend found in this study is not always monotonous (from sighted to visually disabled to totally blind). Especially in the Austrian sample, the students with visual disability are outliers with concern to working memory. One plausible explanation is that not the degree of visual impairment itself is important, but the personal and environmental reaction how to deal with this impairment. However, the general trend shows that cognitive ability seems to be modifiable by visual impairment. Or in other words: Human working memory seems to adapt to special experiences such as blindness which, few would argue, is one of the strongest experiences known.

In fact, our study cannot conclusively distinguish whether and to which degree neuro-biological factors (neuroplasticity, so that brain resources which are otherwise invested in processing visual input can be used for other cognitive processes) or environmental factors (e.g., education adapted to blind persons, the use of braille, receiving similar assistance) lead to improved WMC of persons with visual impairment. Therefore, further studies should differentiate between individuals with visual impairment at birth and visual impairment acquired later in life.

## Conclusion

Based on our findings, it can be ruled out that other factors which are completely independent of visual impairment can account for the clear pattern of effects (WMC increased and VC decreased) found in our study. Especially, as the effects found were controlled for social status, race-ethnicity, and even country, and are in line with other studies showing the same stable pattern of effects across different samples, different decades, different countries, as well as different authors.

Our data also suggest that those differences in working-memory capacity are not on *g*, which could be seen as suggesting they are not genetic or biological. Accordingly, no theory assumes common genetic effects on intelligence and eyesight. The modest number of subtests did not allow the use of multi-group confirmatory factor analysis. So, further research using more subtests is strongly recommended. Nevertheless, our results seem to suggest that the differences in WMC are not genetic or biological.

That family background factors have an impact on children’s cognitive ability measures is not surprising; in a study covering seven countries, including developed and developing countries, the statistically most important factor was ethnicity-migration background followed by parental educational level (Rindermann and Ceci, 2018). Important in our study is, that eyesight still has a robust positive effect on working memory (increasing) and robust negative effect on verbal comprehension (decreasing).

We cannot rule out the possibility that the quality of the schools for the blind was at a higher level than the quality of regular schools. At least, the schools for the blind are comprehensively adapted to the special needs of the visually impaired and the blind. So, current inclusion-based attempts to close such schools and to send students with a visual disability to regular schools, where they usually do not receive the intensive treatment by specialist staff, may endanger the development of children with special needs such as students with visual disability. Here, the current study is of course just one first “piece of evidence”: More extensive research controlling for potentially different quality of schools for students with versus without visual impairment is needed. Finally, meta-analyses should be carried out, including controlling for publication bias.

## Data Availability Statement

The datasets generated for this study are available on request to the corresponding author.

## Ethics Statement

Ethical review and approval was not required for the study on human participants in accordance with the local legislation and institutional requirements. Written informed consent to participate in this study was provided by the participants’ legal guardian/next of kin.

## Author Contributions

HR: conceptualization and methodology and investigation. AA: project administration and writing – original draft and preparation. HR and JN formal analysis and writing – review and editing. All authors contributed to the article and approved the submitted version.

## Conflict of Interest

The authors declare that the research was conducted in the absence of any commercial or financial relationships that could be construed as a potential conflict of interest.
