# Supplementary material for: Does Blindness Boost Working Memory? A Natural Experiment and Cross-Cultural Study
Source: Front Psychol. 2020 Jul 3;11:1571. doi: 10.3389/fpsyg.2020.01571 (PMC7347789; doi:10.3389/fpsyg.2020.01571)
Supplement: Supplementary file 1 [file Presentation_1.pdf]

Supplemental material: G-factor analysis

The first idea of this *g*-factor analysis is that the differences between the sighted and blind students may be higher if they are based on *g*-factor scores instead of subtest means. *g*-factor scores measure traits somewhat more reliably than mere mean scores (Jensen, 1998). The second idea is that if eyesight (visual impairment) shows higher correlations with subtests loading high on a working memory *g* factor then the eyesight effects on intelligence can be seen as more biologically-neurologically based (Jensen, 1998). This indicates neither biological nor environmental roots of visual impairment, but it points to the intelligence effects of visual impairment. Because we have only three WMC and four verbal subtests the analyses are only provisional (i.e., multi-group confirmatory factor analyses are not possible and the results should be interpreted cautiously). Nevertheless, we are well aware that the number of subtests is quite small and therefore results have to be handled with caution.

We took all three working-memory subtests and all four verbal comprehension subtests and computed for each a *g* factor score, so resulting in two *g* factor scores (a WMC-*g* and a VC-*g*). For comparison, we also based the working-memory and verbal comprehension scale averages on all (three, respectively, four) subtests. Then we correlated them with visual impairment. The correlations between the full working-memory scale and visual impairment and between the working-memory *g* factor and visual impairment differ only |.02| (*g* factor correlation slightly higher); for the correlation between verbal comprehension and visual impairment (scale mean vs. *g* factor), the difference is only |.002| (slightly higher for the scale mean). There is no increase in differences between the blind and sighted using a *g* factor.

We continued by correlating the *g*-factor loadings of the subtests with their correlation with visual impairment (for instance, with the differences between the blind and sighted). For working memory, this correlation was positive ($r = +.51$, $k = 3$ subtests), but for verbal comprehension negative ($r = -.22$, $k = 4$ subtests). While the first positive correlation is in line with a genetic-biological causation the second negative one is not. The outcomes of all four *g*-factor analyses combined (differences between the blind and sighted are not on *g*) are not in line with a hypothesis of genetic contributions to the difference between the blind and sighted. This tentatively suggests

that not blindness itself or any possible biological causes for blindness are relevant for changes in intelligence, but a behavioral and cognitive reaction of the person and the environment.
